# Takotsubo Cardiomyopathy Complicating Diabetic Ketoacidosis, Hypothermia and Hypernatremia in a Comatose Patient

**DOI:** 10.7759/cureus.65009

**Published:** 2024-07-20

**Authors:** Nagam AlShehabi, Yusuf Hallak, Umberto Battistin, Hanan Faraji, Malek Othman, Hamza Alkowatli, Mehmood Butt

**Affiliations:** 1 College of Medicine, Mohammed Bin Rashid University of Medicine and Health Sciences, Dubai, ARE; 2 Department of Internal Medicine, HCA Florida Blake Hospital, Bradenton, USA; 3 Cardiology, King's College Hospital Dubai, Dubai, ARE

**Keywords:** comatose, hypernatremia, catecholamine surge, stress-induced cardiomyopathy, myocardial stunning, diabetic ketoacidosis, takotsubo cardiomyopathy

## Abstract

Takotsubo cardiomyopathy (TCM) is a transient wall motion abnormality of the left ventricular apex associated with emotional or physical stress. In the setting of diabetic ketoacidosis (DKA), it is thought to be caused by the compound effect of a catecholamine surge and the noxious effect of acidosis and ketones leading to myocardial stunning. In this report, the first of its kind in the Middle East, we describe the case of a 71-year-old comatose patient, who was being treated for DKA and hypernatremia and was incidentally diagnosed with TCM. We also review 15 case reports of DKA-induced TCM published to date in the literature, many of which had an atypical presentation and good outcomes. Furthermore, we discuss possible risk factors for TCM in our case and supporting literature. It is recommended to maintain increased vigilance and attempt early identification of such conditions in acutely ill patients to prevent life-threatening complications.

## Introduction

Takotsubo cardiomyopathy (TCM) is a transient wall motion abnormality of the left ventricle typically associated with emotional or physical stress [1]. While the pathophysiology of TCM remains unknown, various hypotheses have been discussed including myocardial ischemia, left ventricular outlet tract obstruction, autonomic nervous system dysfunction and microvascular dysfunction [1], with the most postulated pathology being neurohormonal release of catecholamines [2]. In addition, another mechanism suggested is the intricate relationship and interplay between the catecholamines and steroids and the effect of this interplay on myocytes. Numerous case reports showcased the role of steroid administration or excess in precipitating TCM, which is likely explained by the well-studied role of steroid hormones in stimulating the synthesis and potentiating the actions of catecholamines on the myocytes [3]. On the other hand, multiple reports have also demonstrated the triggering of TCM in steroid-insufficient patients. Possible processes explaining this association could be found in adrenalectomized animal models, where the toxicity of catecholamines on the steroid-starved cardiac tissue was demonstrated [4]. Other theories include uncoupling of excitation-contraction and aberrant calcium transport in the steroid-starved myocardial cells [5].

The presenting symptoms range from common ones like chest pain and dyspnea to rare reports of vomiting, palpitations, nausea, syncope, or cardiogenic shock. The most common ECG finding is ST elevation (in 44% of patients) followed by T-wave inversion (in 41%) [6]. Other findings include ST depression (in 8%) and left bundle branch block (LBBB; in 5%) [6]. In addition, patients with TCM characteristically show apical akinesia or hypokinesia and an apical balloon-like dilation pattern associated with preservation of contractility of the heart base [1,2].

The treatment of TCM remains individualized, based on the hemodynamic stability, with the mainstay of treatment being addressing the underlying etiology and the prevention of sympathetic activity by beta-adrenergic blockade [7].

Although there are several studies reporting diabetic ketoacidosis (DKA)-associated TCM, there is a scarcity of literature on the symptomatology of this presentation, especially in comatose patients. As the Middle East has the highest prevalence of diabetes worldwide [8], it is vital to examine the pathophysiology and symptomatology of TCM as it represents a rare but potentially fatal complication of DKA [9].

In this report, we present the case of a 73-year-old comatose woman who presented with DKA, was incidentally found to have ST elevation on cardiac telemetry in the ICU with elevated troponin-I but non-obstructed coronary arteries and was eventually diagnosed with TCM. Our patient is the first case in the Middle East to have DKA-associated TCM and the first reported DKA-associated TCM case to have associated hypernatremia.

## Case presentation

A 71-year-old bedridden, cachectic Middle Eastern female was brought by her relatives to the emergency department (ED) with a three-day history of progressive fatigue, poor appetite, loss of sensorium, nausea and vomiting. Her relatives denied any fever or shortness of breath, but reported a one-day history of a mild non-productive cough along with abdominal pain for which she was given pain medications and discharged at another emergency department. Patient mobilizes with assistance to the bathroom and eats well orally. Her past medical history is notable for hypertension (on 5mg lisinopril once daily [OD]), hypercholesterolemia (on atorvastatin 20 mg OD) and poorly controlled type 2 diabetes mellitus (home glucose monitoring ~ 200mg/dl) on oral medications (on empagliflozin 10mg OD, sitagliptin/metformin 50/1g OD, gliclazide modified release 60mg OD). Patient is also on daily calcium supplements (on calcium carbonate 600 mg OD). Surgical history is significant for abdominal surgery three years ago.

On initial evaluation, she was lethargic and severely drowsy with no response to verbal commands, with a Glasgow Coma Scale (GCS) of 6/15 (E4V1M1), pupils equal and reactive to light, and signs of severe dehydration. The patient was hypothermic (34.7◦C) with blood pressure of 156/59 mmHg, heart rate of 114/min, respiratory rate of 36/min, and 96% O2 saturation on room air. Peripheries were cold and clammy. Abdomen was soft and nontender and chest was clear to auscultation bilaterally. The remainder of the examination was unremarkable.

Laboratory investigations identified severe DKA (random glucose level of 450mg/dl, serum bicarbonate level of 2 mmol/l and pH of 6) as well as severe hypernatremia of 170 mmol/l which were treated in ED with 2 L of sodium chloride 0.9% followed by 150ml/hr infusion and free water nasogastric flushes, 100ml of bicarbonate, 20mg enoxaparin SQ, 10U of insulin glargine followed by 5U/hr insulin infusion, pantoprazole and 2g of ceftriaxone intravenously. Labs also showed mild acute kidney injury (AKI), neutrophilic leukocytosis, potassium of 2.4 mmol/l and ketonuria. Chest X-ray (CXR) was unremarkable. Patient was then shifted to the High Dependency Unit for further treatment where her leukocytosis, creatinine, acidosis and electrolytes responded to treatment and were all corrected.

**Figure 1:**
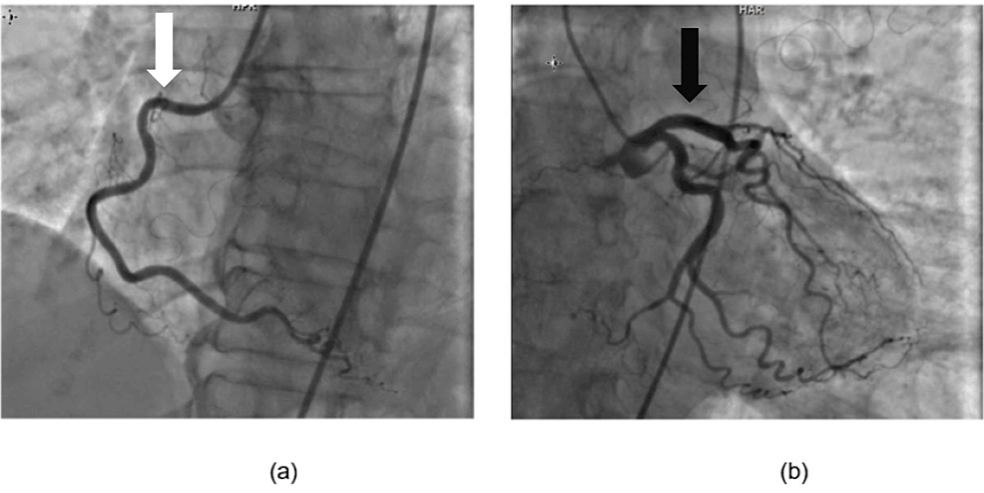
Coronary angiography showing no significant coronary artery disease in RCA (a) (White arrow) and LCA (b) (Black arrow) RCA: Right coronary artery, LCA: Left coronary artery

**Figure 2:**
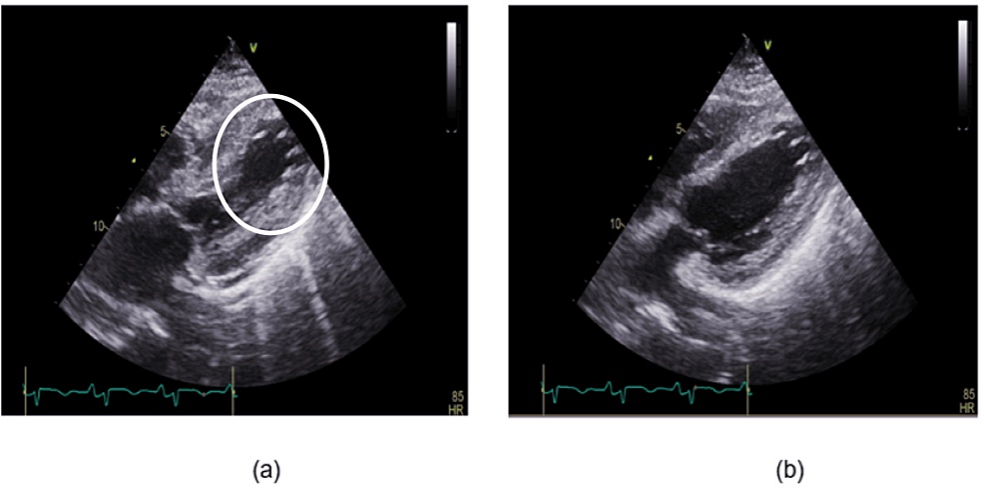
Echocardiogram showing ballooning of the apex observed during systole (a) (White circle) compared to diastole (b)

Incidentally, the patient was found to have ST elevations of the inferolateral leads consistent with an inferolateral ST-elevation myocardial infarction (STEMI). The patient was therefore taken to the Cath Lab for coronary angiography, which revealed right coronary artery (RCA)-dominant circulation and no significant obstructive coronary artery disease (Figure 1). Subsequently, troponin studies and an echocardiogram were ordered, which revealed a troponin-I of 10.61 ng/mL and echocardiographic features suggestive of TCM with ejection fraction (EF) of 35-40% (Figure 2). Patient was then started on aspirin, beta-blocker and high-dose statin. Her troponin then started to trend downward.

The hospital course was complicated by a persistently low GCS despite correction of acidosis and electrolytes, which was concerning for cerebral edema or cerebral venous thrombosis. Magnetic Resonance Venography (MRV) was negative for venous thrombosis, however brain Magnetic Resonance Imaging (MRI) identified subcortical white and gray matter infarcts with pan-ventriculomegaly, postulated to be from normal pressure hydrocephalus or possible leukomalacia and subcortical infarcts. Electroencephalography (EEG) also demonstrated diffuse slowing of brain activity in theta>delta range suggesting encephalopathy. Lumbar puncture excluded meningitis. Nerve conduction study was also significant for a moderately severe length-dependent sensorimotor axonal neuropathy consistent with diabetic polyneuropathy or critical illness polyneuropathy. Patient was thus started on thiamine replacement and a percutaneous endoscopic gastrostomy (PEG) tube was inserted due to low caloric intake as well as swallowing dysfunction. Ultrasound Kidney-Ureter-Bladder showed irregular echogenicities consistent with blood clots. Computed Tomography Abdomen showed fatty liver with old granuloma, dorsolumbar spine degeneration and bilateral pleural effusion with atelectasis.

Following her prolonged hospital stay due to nosocomial infections (e.g. aspiration pneumonia, wound infection, and PEG site abscess), the patient was eventually discharged on Day 37 with instructions for home nursing care and physiotherapy with special attention to the nutritional requirements and PEG tube maintenance. Medications on discharge included a five-day course of ceftriaxone 1gm twice daily (BID), pantoprazole 40 mg OD via PEG tube, two tablets of calcium carbonate BID via PEG tube, insulin glargine subcutaneously (SQ) 26 Units (U) OD, insulin NovoRapid SQ 14U pre-feed and 10U during feeds BID and Glucerna 80ml/hr for 20hrs. Since discharge, the patient has had recurrent admissions (UTI, PEG leak, pneumonia) with the last admission being due to seizures. There was no follow-up echocardiogram or work-up related to her TCM diagnosis.

## Discussion

In this paper, we describe the case of TCM in a comatose patient, with underlying DKA, hypothermia and hypernatremia. TCM, also known as stress-induced cardiomyopathy, apical ballooning syndrome or “broken heart” syndrome, is a reversible cardiomyopathy characterized by left ventricular dysfunction typically with apical hypokinesia and ballooning with a clinical presentation mimicking acute coronary syndrome (ACS; ST elevation, T wave inversion, Q wave abnormalities on ECG as well as raised cardiac enzymes) in the absence of angiographic evidence of coronary occlusion [2,10]. Four classifications based on pattern of wall motion abnormalities have been reported: Takotsubo type (most common), reverse Takotsubo type, mid-ventricular type, and localized type [11]. Another classification divided TCM into apical (most common), midventricular, basal, or focal subtypes [6].

Patients often present in the context of emotional and physical stress [2]. Patients with hyperlipidemia, pulmonary hypertension, subarachnoid hemorrhage, migraines, hyperthyroidism, collagen vascular diseases, anxiety states, and stress have higher odds of developing TCM [8]. Mayo Clinic’s modified criteria of TCM comprise transient left ventricular systolic dysfunction, regional wall motion abnormalities beyond a single coronary artery, non-obstructive coronary angiography, new ECG changes or elevated cardiac enzymes, and ruling out pheochromocytoma or myocarditis [12]. Furthermore, TCM typically resolves within two months [13].

Our patient is the first case of DKA-associated TCM in the Middle East. The literature examining this association has been reviewed (Table 1) [9,14-27] and showed a total of 15 cases. Most cases have been reported in the USA, with the first case being reported by a Taiwanese hospital in 2007 [14]. The ages of reported cases ranged between 18 and 81 years with an average of 53.5 years and median of 57 years. Our case serves as the second oldest case. Male cases percentage was 18.75% in our literature review, which conforms to the previously reported male cases percentage of 9.4-22.8% [28].

**Table 1:** Literature review on the association between DKA and Takotsubo or Stress Cardiomyopathy up to April, 2023 T2DM= type 2 diabetes mellitus; Temp= temperature; BP= blood pressure; HR= heart rate; Na= serum sodium; Glu= serum glucose; pH= power of hydrogen; HCO3= serum bicarbonate; CXR= chest x-ray; CK: creatine kinase; CKMB= creatine kinase myocardial band; ECG= electrocardiogram; Echo= echocardiogram; EF= ejection fraction; DKA= diabetic ketoacidosis; T1DM= type 1 diabetes mellitus; ARDS= adult respiratory distress syndrome; SO2= oxygen saturation; CT= computed tomography; LP= lumbar puncture; HOCM= hypertrophic obstructive cardiomyopathy; AF= atrial fibrillation; DM= diabetes mellitus; SAM= systolic anterior motion; WBC= white blood cells count; LV= left ventricle; AKI= acute kidney injury; K+= serum potassium; EEG= electroencephalogram; MRI= magnetic resonance imaging; ECMO= extracorporeal membrane oxygenation

Author	YOP	Title	Country	Clinical picture	Diagnosis	Hypernatremia	Hypothermia	Treatment and Complications
Cheng-Hui Lin, et al. [14]	2007	Ampulla Cardiomyopathy (Takotsubo Cardiomyopathy) in A Patient with Diabetic Ketoacidosis A Case Report	Taiwan	71-year-old woman with history of T2DM and hypertension presented with mild chest tightness and dyspnea. T = 38.5°C, BP = 93/55 and HR = 131/min. Labs showed Na = 134.4 mmol/l, Glu = 432 mg/dl, pH = 7.376, HCO3 = 17.8 mEq/l, ketonemia and pyruria. CXR revealed increased interstitial markings bilaterally. Urine culture grew Escherichia coli.	CK = 651 U/l. CKMB = 33.7 U/l. Troponin-I = 5.077 ng/dl. ECG showed ST-segment depression in leads III and aVF as well as ST-segment elevation in leads I, aVL, and V1-6. Following ECGs showed giant inverted T-wave and QT prolongation on Day 2. Echo showed akinesis of the anterior wall, apex, distal septum, and distal lateral wall as well as hypokinesis of the mid to distal inferior wall and posterior wall. Coronary angiography revealed normal coronaries. Left ventriculogram showed apical ballooning and akinesis with EF of 58%.	No	No	DKA protocol, heparin, aspirin, isosorbide dinitrate and antibiotics were started. The symptoms improved and echo showed recovery of wall-motion abnormalities with EF 63% two weeks later. Patient was stable on follow-up.
Sudip Nanda, et al. [15]	2009	Stress cardiomyopathy - a unique presentation of diabetic ketoacidosis	USA	46-yr-old woman with T1DM on insulin pump who ran out of insulin and presented with nausea, vomiting and epigastric abdominal pain. Her labs showed Na = 132 mmol/l, HCO3 = 4 mmol/l, Glu = 674 mg/dl.	Troponin = 7.75 ng/ml and CK = 652 U/l. ECG showed ST-elevations in both anterior leads (V2–V6) and inferior leads (II, III and aVF). Echo showed left anterolateral wall hypokinesia and severe apical akinesia and ballooning. EF was 30%. Coronary angiography revealed normal coronaries.	No	Not mentioned	DKA protocol was initiated and DKA rapidly resolved. 12-day follow-up echo revealed resolution of abnormalities. The patient was re-admitted 4 weeks later with DKA.
Yoichi Katayama, et al. [16]	2013	A case of Takotsubo cardiomyopathy induced by accidental hypothermia and diabetic ketoacidosis	Japan	59-year-old woman presented with lethargy, drowsiness and syncope with history of chest pain 1 day prior. Temp = 30.9°C, Glu = 1018 mg/dl and pH = 6.78 with ketonuria.	CK = 1951 U/l and Troponin I = 7.94 ng/ml. ECG demonstrated a J wave (V4-6) and ST-segment elevation in (V3-5), II, III and aVF leads. Echo revealed left ventricular wall systolic dysfunction at the apex with hypercontraction of the basal segment. Coronary angiography revealed normal coronaries.	Not mentioned	yes	Patient was diagnosed with new onset T1DM. Patient was intubated and started on warmed crystalloid fluids, DKA protocol and antibiotics. Patient was then stabilized with an intra-aortic balloon pump and inotropes. ECG and echo findings resolved on day 2 and 13, respectively.
Wei-Tsung Wu, et al. [9]	2014	A Case of Takotsubo Cardiomyopathy Precipitated by Thyroid Storm and Diabetic Ketoacidosis with Poor Prognosis	Taiwan	81-year-old woman with T2DM, hypertension and an stroke presented with palpitation, chest tightness, abdominal fullness, vomiting and diarrhea on a background of anxiety and excessive sweating for the last three months. Temp = 38.5 °C, BP = 110/60 and HR = 146/min. Labs showed Glu = 566 mg/dl, Serum Osmolarity = 339 mOsm/l, ketonemia, pH = 7.16 and HCO3 = 8.3 mmol/l	CKMB = 17.5 ng/ml and Troponin = 8.75 ng/ml. ECG revealed sinus tachycardia with ST elevation over V2-V4. Echo revealed EF= 35.4% and apical hypokinesia/akinesia with ballooning. Coronary angiography revealed normal coronaries.	Not mentioned	No	DKA protocol was started but the patient didn’t improve. Thereafter, thyroid storm was diagnosed. Follow-up echo revealed improvement of apical ballooning and EF to 59.2%. The patient finally died on the 8th day due to ARDS progression.
Andrew S Lane, et al. [17]	2015	Diabetic ketoacidosis due to fulminant type 1 diabetes: A rare subtype of type 1 diabetes leading to unusual sequelae	Australia	Previously healthy 18-year-old man presented with drowsiness, lethargy, vomiting and tachypnoea. Temp = 34.0℃, HR = 130/min, BP = 105/48 and SO2 was 90% on 6 L of O2 with abdominal guarding and neck stiffness. His labs showed pH = 7.13, HCO3 = 9 mmol/, creatinine = 4.29 mg/dl, normal lipase, ketonemia, Glu = 900 mg/dl (peak 1800 mg/dl) and lactate = 5 mmol/l. CT brain was unremarkable and LP showed high glucose only. CXR and chest CT showed subcutaneous emphysema and pneumomediastinum with normal gastrograffin study.	High-sensitivity Troponin = 61 ng/l (peaked to 1856 ng/l) and CK = 26784 U/l. Initial ECG showed sinus tachycardia. A second ECG showed ST elevation in leads II, III and aVF with right bundle branch block. Echo showed global left ventricular hypokinesis with EF= 15%, apical akinesis/dyskinesis and severely reduced right ventricular systolic function. Viral and autoimmune workup of cardiomyopathy was negative.	Not mentioned	Yes	DKA protocol and inotropes were started and patient was intubated and transferred to ICU. Patient was diagnosed with new-onset fulminant T1DM. Patient recovered with EF function normalizing at day 5. He was discharged at day 10.
Ayla Gordon, et al. [18]	2017	DKA-Induced Takotsubo Cardiomyopathy in Patient with Known HOCM	USA	66-year-old man with history of hypertension, HOCM and Meniere’s disease on steroids presented with syncope. HR = 116/min and BP = 108/74. Labs revealed pH = 7.12 and Glu = 526 mg/dl. An old echo documented signs of HOCM with SAM, an intraventricular gradient of 32 mmhg and EF = 75%.	Troponin = 13.8 ng/ml with ECG showing AF and ST elevations in leads V3–V6, II, III and aVF. Coronary angiography demonstrated normal coronaries, EF = 20% as well as anterolateral, apical, and inferoapical dyskinesis and basal anterior and basal inferior wall hyperkinesis. Echo showed apical hypokinesis and EF = 35–40%.	Not mentioned	Not mentioned	New diagnosis of DM was made with no evident end-organ complications. Patient was started on DKA protocol, anticoagulation, carvedilol and diltiazem for rate control of AF. DKA was controlled by day 4 and repeat echo on day 5 showed documented EF= 45% and resolution of motion abnormalities.
Jed H. Meyers, et al. [19]	2017	Takotsubo Cardiomyopathy in Association with DKA in A Blind Pump Patient	USA	70-year-old blind woman with long-standing T1DM on insulin pump who likely mistakenly filled her pump reservoir with air presented with loss of consciousness. Temp = 34.4◦C, BP = 69/49 and HR = 88/min. Labs showed Glu = 1290 mg/dl, Na = 131 mmol/l, HCO3 = 12 mmol/l, ketonemia, lactate = 56 mmol/l, creatinine = 3.96 mg/dl and WBC = 25.1 x10^3^/µL.	CK = 943 U/l and Troponin T = 0.12 ng/ml. ECG showed septal ST depression. Echo showed a left ventricle of normal size with a large apical aneurysm on day 4.	No	Yes	DKA protocol and fluid resuscitation were started. Repeat echo 18 days later showed resolution of the aneurysm. Patient was discharged home on insulin.
Muzammil Khan, et al. [20]	2018	A Case of Euglycemic Diabetic Ketoacidosis due to Canagliflozin Complicated by Takotsubo Cardiomyopathy	USA	56-year-old woman with history of T2DM on metformin, exenatide, glipizide and recently canagliflozin presented with acute confusion. Temp = 36.5◦C and HR = 110/min. Labs showed creatinine = 1.4 mg/dl, bicarbonate <5 mEq/l, ketonemia, Glu = 170 mg/dl, pH = 6.94, WBC = 20 x 10^3^/µL and lactate = 2.7 mmol/l. CT head without contrast showed no acute pathology and CXR showed left lower lobe consolidation.	Troponin I = 0.05 ng/ml (peaking at 0.74 ng/ml). CK and CK-MB were normal. ECG revealed sinus tachycardia and old left bundle branch block. Echo 12 hours after the cardiac arrest revealed newly depressed EF 21% (61% 6 months ago), normal LV size and LV wall thickness, and septal wall motion abnormality. Patient refused to undergo cardiac catheterization	No	No	Patient was transferred to ICU, intubated, started on IV fluids, DKA protocol and antibiotics. Patient went into cardiac arrest during central line insertion. DKA and AKI resolved and patient was extubated. EF improved to 55% a week later and her troponin plateaued at 0.63 ng/ml with no new ECG changes.
C. R. Pendkar, et al. [21]	2018	Diabetic Ketoacidosis and Takotsubo Cardiomyopathy in Hereditary Hemochromatosis	USA	57-year-old woman with history of hereditary hemochromatosis on monthly phlebotomies presented with abdominal pain, vomiting, dizziness, dyspnea and chest pain. Patient was tachycardic. Labs showed Glu = 345 mg/dl, acidosis, ketonuria, mild transaminitis, leukocytosis and negative urine drug screen.	Troponin was elevated. ECG showed sinus tachycardia. Echo showed acute severe left ventricular systolic dysfunction with an EF of 20-25% and multiple segmental abnormalities.	Not mentioned	Not mentioned	Patient was admitted to ICU and received DKA protocol, diuretics, and carvedilol. Echo 1 week later showed EF of 40-45%.
Mohammed Mhanna, et al. [22]	2020	A Case of Takotsubo Cardiomyopathy Triggered by Diabetic Ketoacidosis and Hypothermia	USA	A 50-year-old woman with history of T1DM presented with lethargy. Temp = 32.2◦C, BP = 74/64 and HR = 96/min. Labs showed Glu = 1637 mg/dl, pH = 6.8, ketonemia, HCO3 = 2.7 mmol/l, creatinine = 4.59 mg/dl and K+ =7.6 mmol/l. Head CT and CXR were unremarkable.	On admission, Troponin = 0.21 ng/ml, ECG showed transient accelerated idioventricular rhythm with diffuse ST depression, and echo revealed EF: 60%-65%. Later, troponin levels increased to 20 ng/ml, and ECG showed ST-elevation in leads II, III, aVF, and V3-6. Echo showed EF of 25% with globally hypokinetic LV. Coronary angiography revealed normal coronaries with a severely reduced cardiac index. Pulmonary CT angiography didn’t reveal a pulmonary embolism.	Not mentioned	Yes	Patient was transferred to ICU, intubated, and started on DKA protocol and hemodialysis. On Day 2 of admission, the patient developed cardiogenic shock resistant to inotropes. Patient was started on beta-blockers and ACE inhibitor, extubated and fitted with a wearable life vest/ defibrillator. 1-month Follow-up Troponin was 0.02 ng/ml, ECG normalized, and echo showed improved EF and ventricular motion.
Khaing Khaing Htwe, et al. [23]	2020	Association Of Diabetic Ketoacidosis with Takotsubo Cardiomyopathy	USA	57-year old woman with poorly controlled T2DM presented with nausea and vomiting. She was normotensive with HR = 114/min. Labs showed Glu = 560 mg/dl, HCO3 = 3 mmol/l, pH = 6.98 with ketonuria. CXR was clear.	Troponin was elevated. ECG showed ST elevation in V3-V6 and Q waves in V1-V2. Echo showed EF = 40-45% with anteroseptal and apical akinesia. Coronary angiography revealed normal coronaries.	Not mentioned	Not mentioned	DKA protocol, heparin and dual antiplatelet therapy were started. DKA was rapidly corrected. Cardiac MRI a week later showed near normalization of EF and no late gadolinium enhancement. She was discharged on ACE inhibitor, beta blocker, and insulin.
Mary Dickow, et al. [24]	2021	A Case Report of DKA-Induced Takotsubo Cardiomyopathy in A Stiff Person Syndrome on IVIG	USA	68 year-old woman with history of AF, T1DM and stiff man syndrome on monthly IVIG presented with DKA. Labs were not mentioned.	ECG demonstrated AF with rapid ventricular response. Echo showed EF of 30- 35% and apical, distal anteroseptal, and basal inferoseptal hypokinesis. Cardiac catheterization demonstrated minimal vessel disease with mild apical hypokinesis	Not mentioned	Not mentioned	Not mentioned
Ahmed Abbas, et al. [25]	2022	Diabetic Ketoacidosis-Induced Cardiomyopathy and Reversible Dialysis-Dependent Renal Failure with Successful Outcome: A Report of a Rare Case	USA	37-year-old man with a history of poorly controlled T2DM on insulin presented with unconsciousness, bloody vomiting and tender left upper abdomen. BP = 70/40 and HR = 110/min. Labs showed HCO3 = 13 mmol/l, pH = 7.25, Glu = 2088 mg/dl, ketonemia, Lactate = 9 mmol/l, creatinine = 7.6 mg/dl, Hypertriglyceridemia, hypophosphatemia and lipase = 2900 U/l. Abdominal CT showed no signs of pancreatitis. Septic work-up, EEG, urine drug screen and MRI brain were negative.	CK = 2200 U/l and high-sensitivity Troponin = 18 ng/l (peaked at 505). ECG demonstrated sinus tachycardia with right axis deviation and repolarization abnormalities. Echo showed diffuse global hypokinesis with EF of 30%.	Not mentioned	Not mentioned	The patient was intubated and started on DKA protocol, IV resuscitation, antibiotics, inotropes, and hemodialysis. Echo showed an improved EF of 60% with normal ventricular motion on day 14. Patient was discharged on Insulin 23 days later.
Julieta Lacey [26]	2022	1502: Unexpected Heartbreak: A Case of DKA Complicated by Stress Cardiomyopathy and Cardiogenic Shock	USA	A previously healthy 25-year-old woman with acute encephalopathy. Labs showed Glu = 614 mg/dl, HCO3 = 2 mmol/l	Troponin = 1.68 ng/ml. Echo showed left ventricular systolic dysfunction with EF of 25% and wall motion abnormalities of the basal and mid-anterior wall and septum. Endomyocardial biopsy was negative for myocarditis.	Not mentioned	Not mentioned	Patient decompensated hemodynamically despite medical management and was started on Venoarterial ECMO and decannulated on day 7. Repeat echocardiogram was normal on 10 day.
Riley Gurreri, et al. [27]	2023	Cardiogenic shock secondary to stress-induced cardiomyopathy precipitated by severe diabetic ketoacidosis	USA	Previously healthy 25-year-old woman presented with loss of consciousness, weakness and lethargy. HR = 113/min and BP =113/68. Initial Glu was incalculably high, pH	Troponin = 0.27 ng/ml, peaking at 4.68 ng/ml. BNP = 329. Initial ECG demonstrated sinus tachycardia with borderline prolonged QT. Echo showed EF of 20–25% with basal and mid anterior wall motion abnormalities as well as basal and mid-septal abnormalities. On day 6, coronary angiography revealed normal coronaries. Viral and autoimmune work-up was negative for cardiomyopathy. Right ventricular biopsy was negative.	Not mentioned	Not mentioned	Patient was transferred to ICU and started on DKA protocol, aggressive resuscitation, sodium bicarbonate and electrolytes but patient developed cardiogenic shock, AKI, polyglandular failure and polyradiculoneuropathy necessitating inotropes, ECMO and IVIG. Patient was diagnosed with T1DM. Repeat echo showed EF of 50% with global hypokinesia on day 6. Patient improved and was discharged by day 23 on aspirin, ACE inhibitor and beta-blocker.

There is scarcity of literature on the symptomatology of DKA-associated TCM, especially in comatose patients where typical symptoms of TCM that tend to mimic ACS cannot be expressed. Our patient, due to her persistently low GCS, was unable to express the classical ACS-like symptoms of TCM (e.g. sudden chest pain, shortness of breath, syncope, etc.) and presented instead with traditional DKA symptoms (fatigue, poor appetite, loss of sensorium, nausea, vomiting, abdominal pain) with TCM incidentally discovered on ECG without hemodynamic instability. Meanwhile, our literature review revealed that the symptomatology was divided almost equally into DKA-symptoms [15,23,24,26], TCM-symptoms [14,18-20,22,27] and mixed-symptoms groups (Table 1) [15-17,21,25]. It should be noted that DKA-specific symptoms and classic cardiac TCM symptoms can overlap, necessitating a high index of suspicion in patients with risk factors for TCM.

Our case was treated for TCM with aspirin, beta-blocker, and high-dose statin, with no long-term TCM-specific treatment. Our literature review revealed that treatment approaches to DKA-associated TCM included: supportive treatment, antiplatelets, anticoagulants, diuretic, nitrates, beta-blockers, inotropes, mechanical support and extracorporeal membrane oxygenation system (ECMO) (Table 1). It should be noted that despite inotropes, specifically beta agonists, being relatively contraindicated in TCM [29], four studies have included inotropes as part of DKA-associated TCM management [16,17,22,25]. However, further studies are needed to elucidate the indications, benefits, and risks of inotropes in DKA-associated TCM cases complicated by cardiogenic shock.

While our case developed significant morbidity shown by the long hospital stay with complicating infections and persistent encephalopathy with recurrent seizures, almost all the review cases had good outcomes, except for one reported case by Wu et al. [9] who passed away due to adult respiratory distress syndrome (ARDS) progression after resolution of TCM.

As can be seen by our literature review, the association between TCM and DKA has rarely been reported. Theories included those posited by Nanda et al. [15], who explained that severe acidosis’s effect on myocardial calcium channels and myocardial apoptosis along with ketonemia’s effect on glucose utilization; Mhanna et al. [22] who suggested that catecholamine excess was the culprit in the mechanism of TCM in the setting of DKA; and Gordon et al. [18] who postulated that catecholamine surge compounded with ketoacidosis contributes to dysfunction myocardial calcium channels, leading to myocardial stunning and resultant TCM. Of note, our literature review showed that some cases of DKA presented with TCM despite exhibiting mild [14] or moderate [19,25] acidosis.

On the other hand, it has been argued that due to autonomic neuropathy, diabetes decreases both direct and indirect adrenergic stimulation of the myocardium, with an expected decrease in the occurrence of TCM, and would thus exert a “cardioprotective effect”. Madias et al. [30] demonstrated that in patients with TCM, the prevalence of diabetes mellitus is lower than the general population, possibly due to the cardioprotective effect of autonomic neuropathy. This prompts further investigations to consolidate the understanding of the underlying pathophysiology of TCM in diabetic patients.

Multiple cases reported hypothermia in cases of DKA-associated TCM [16,17,19,22], with most of the cases having concomitant shock [16,19,22]. Hypothermia can also be caused by hypothyroidism, adrenal insufficiency, neurological pathologies, certain substances/medications [31] as well as DKA due to unavailability of glucose as a substrate for heat generation [32]. Hypothermia has also been described to contribute to catecholamine release when coupled with acidosis [18].

While multiple cases described the association between hyponatremia and TCM, our case is only the third to report hypernatremia in TCM [33,34]. We hypothesize that hypernatremia could serve as an additional stressor by increasing catecholamine release and decreasing left ventricular inotropy [35]. Hypokalemia has also been reported to be associated with TCM [36-37]. In addition, encephalopathy is a well-reported cause of TCM [38]. This patient’s major risk factors, namely hypertension and type 2 diabetes, are known to predispose to cardiac remodeling, which in the setting of acute illness such as DKA and hypernatremia, may precipitate sudden cardiac decompensation. We can postulate that cachexia, severe electrolyte derangements, and DKA can impact the sympathetic nervous system, and this could lead to TCM or predispose patients to develop TCM in the presence of a catecholamine surge.

This study highlights the importance of considering the diagnosis of TCM in patients presenting with acute illness even if they have no comorbidities that would provide a causal link, and also emphasizes the relevance of continuous telemetry and a low threshold for ordering targeted cardiac investigations in patients undergoing physical and psychological stress such as DKA.

Our study has certain limitations. Firstly, our literature review was derived from case reports rather than cohort studies or experimental studies, limiting the conclusions derived from our review. Secondly, despite careful searches, we have probably not included all reported cases of DKA-associated TCM. However, our review is the most complete review of all published DKA-associated TCM cases to date.

## Conclusions

This case study describes a rare instance of TCM in a comatose patient with DKA, hypothermia, and hypernatremia. Despite the patient’s low GCS preventing the expression of typical TCM symptoms, incidental ECG findings led to the diagnosis, underscoring the importance of proactive cardiac monitoring in similar scenarios. Our literature review revealed only 15 reported cases of DKA-associated TCM, with this case being the second oldest and one of the few involving hypernatremia. Several pathophysiological mechanisms explaining the cause of TCM have been postulated, including a catecholamine surge. However, this effect is compounded by the deleterious effects of ketoacidosis on the myocardium in the setting of DKA, leading to myocardial stunning. This case study and literature review emphasise the need for further research to understand the pathophysiology and optimize management strategies for TCM in the context of severe metabolic stressors like DKA.

## References

[1] Komamura K, Fukui M, Iwasaku T, Hirotani S, Masuyama T (2014). Takotsubo cardiomyopathy: pathophysiology, diagnosis and treatment. World J Cardiol.

[2] Gianni M, Dentali F, Grandi AM, Sumner G, Hiralal R, Lonn E (2006). Apical ballooning syndrome or takotsubo cardiomyopathy: a systematic review. Eur Heart J.

[3] Seleznev IM, Martynov AV (1982). Permissive effect of glucocorticoids in catecholamine action in the heart: possible mechanism. J Mol Cell Cardiol.

[4] Cleghorn RA (1983). Cardiovascular failure in experimental adrenal insufficiency: a historical revival. Perspect Biol Med.

[5] Narayanan N (1983). Effects of adrenalectomy and in vivo administration of dexamethasone on ATP-dependent calcium accumulation by sarcoplasmic reticulum from rat heart. J Mol Cell Cardiol.

[6] Templin C, Ghadri JR, Diekmann J (2015). Clinical features and outcomes of takotsubo (stress) cardiomyopathy. N Engl J Med.

[7] Palecek T, Kuchynka P, Linhart A (2010). Treatment of Takotsubo cardiomyopathy. Curr Pharm Des.

[8] (2023). IDF Diabetes Atlas. https://www.diabetesatlas.org.

[9] Wu WT, Hsu PC, Huang HL, Chen YC, Chien SC (2014). A case of takotsubo cardiomyopathy precipitated by thyroid storm and diabetic ketoacidosis with poor prognosis. Acta Cardiol Sin.

[10] Potu KC, Raizada A, Gedela M, Stys A (2016). Takotsubo cardiomyopathy (broken-heart syndrome): a short review. S D Med.

[11] Kazakauskaitė E, Jankauskas A, Lapinskas T, Ordienė R, Ereminienė E (2014). Takotsubo cardiomyopathy: the challenging diagnosis in clinical routine. Medicina (Kaunas).

[12] Prasad A, Lerman A, Rihal CS (2008). Apical ballooning syndrome (tako-tsubo or stress cardiomyopathy): a mimic of acute myocardial infarction. Am Heart J.

[13] Akashi YJ, Nakazawa K, Sakakibara M, Miyake F, Koike H, Sasaka K (2003). The clinical features of takotsubo cardiomyopathy. QJM.

[14] Lin C, Chen C-C, Tsai M-K, Wang Y-C, Chang S, Chang W-T (2007). Ampulla cardiomyopathy (takotsubo cardiomyopathy) in a patient with diabetic ketoacidosis: a case report. Semantic Scholar.

[15] Nanda S, Longo S, Bhatt SP, Pamula J, Sharma SG, Dale TH (2009). Stress cardiomyopathy - a unique presentation of diabetic ketoacidosis. Ann Clin Biochem.

[16] Katayama Y, Hifumi T, Inoue J, Koido Y (2013). A case of takotsubo cardiomyopathy induced by accidental hypothermia and diabetic ketoacidosis. BMJ Case Rep.

[17] Lane AS, Champion B, Orde S, Dravec D (2015). Diabetic ketoacidosis due to fulminant type 1 diabetes: a rare subtype of type 1 diabetes leading to unusual sequelae. J Intensive Care Soc.

[18] Gordon A, LaCapra G, Roberti R (2017). DKA-induced takotsubo cardiomyopathy in patient with known HOCM. Case Rep Crit Care.

[19] Meyers JH, Hirsch IB (2017). Takotsubo cardiomyopathy in association with DKA in a blind pump patient. AACE Clin Case Rep.

[20] Khan M, Khalid S, Marwat A, Mehmood H (2018). A case of euglycemic diabetic ketoacidosis due to canagliflozin complicated by takotsubo cardiomyopathy. Am J Med Case Rep.

[21] Pendkar CR, Shamian B, Shankar S, Saradna A (2018). Diabetic ketoacidosis and takotsubo cardiomyopathy in hereditary hemochromatosis. Crit Care Case Rep.

[22] Mhanna M, Beran A, Srour O, Ghazaleh S, Elzanaty A (2020). A case of takotsubo cardiomyopathy triggered by diabetic ketoacidosis and hypothermia. Cureus.

[23] Htwe KK, Patel P, Soe HM (2020). Association of diabetic ketoacidosis with takotsubo cardiomyopathy. Chest J.

[24] Dickow M, Chaudhry S, Khanam V, Goel M, Kulairiet Z (2021). A case report of DKA-induced takotsubo cardiomyopathy in a stiff person syndrome on IVIG. J Am Coll Cardiol.

[25] Abbas A, Patel N, Kazmi R, Mirza N, Miller R, Correia J (2022). Diabetic ketoacidosis-induced cardiomyopathy and reversible dialysis-dependent renal failure with successful outcome: a report of a rare case. Cureus.

[26] Lacey J (2022). Unexpected heartbreak: a case of DKA complicated by stress cardiomyopathy and cardiogenic shock. Crit Care Med.

[27] Gurreri R, Poommipanit P, Alghamdi A (2023). Cardiogenic shock secondary to stress-induced cardiomyopathy precipitated by severe diabetic ketoacidosis. Oxf Med Case Reports.

[28] Murakami T, Komiyama T, Kobayashi H, Ikari Y (2022). Gender differences in Takotsubo syndrome. Biology (Basel).

[29] Madias JE (2021). Takotsubo cardiomyopathy: current treatment. J Clin Med.

[30] Madias JE (2016). Low prevalence of diabetes mellitus in patients with Takotsubo syndrome: A plausible 'protective' effect with pathophysiologic connotations. Eur Heart J Acute Cardiovasc Care.

[31] Larach MG (1995). Accidental hypothermia. Lancet.

[32] Sheikh AM, Hurst JW (2003). Osborn waves in the electrocardiogram, hypothermia not due to exposure, and death due to diabetic ketoacidosis. Clin Cardiol.

[33] Pruthi S, Kobrossi S, Bartaula R, Chaudhuri D (2017). The misleading electrocardiogram - midventricular Takotsubo masquerading as anterior wall STEMI. Am J Emerg Med.

[34] Hong J, Glater-Welt LB, Siegel LB (2014). Takotsubo cardiomyopathy in a 23 months-old following traumatic brain injury. Ann Pediatr Child Health.

[35] Lenz K, Gössinger H, Laggner A, Druml W, Grimm G, Schneeweiss B (1986). Influence of hypernatremic-hyperosmolar state on hemodynamics of patients with normal and depressed myocardial function. Crit Care Med.

[36] Finsterer J (2019). Recurrent seizure-triggered Takotsubo associated with hypokalemia and hypomagnesemia. Seizure.

[37] Zoltowska DM, Agrawal Y, Kalavakunta JK (2018). Can aldosterone break your heart? Takotsubo cardiomyopathy in a patient with newly diagnosed primary aldosteronism. BMJ Case Rep.

[38] Yamaguchi H, Nagase H, Yoshida S (2019). Acute encephalopathy with biphasic seizures and late reduced diffusion accompanied by Takotsubo cardiomyopathy. Brain Dev.

